# Personal Involvement Moderates Message Framing Effects on Food Safety Education among Medical University Students in Chongqing, China

**DOI:** 10.3390/ijerph15092059

**Published:** 2018-09-19

**Authors:** Li Bai, Zhengjie Cai, Yalan Lv, Tingting Wu, Manoj Sharma, Zumin Shi, Xiaorong Hou, Yong Zhao

**Affiliations:** 1 School of Medical and Information, Chongqing Medical University, Chongqing 400016, China; 2017111298@stu.cqmu.edu.cn (L.B.); yalanlv@126.com (Y.L.); 2 School of Public Health and Management, Chongqing Medical University, Chongqing 400016, China; m18324184027@163.com (Z.C.); m17749945967@163.com (T.W.); 3 Behavioral & Environmental Health, School of Public Health, Jackson State University, Jackson, MS 39213, USA; manoj.sharma@jsums.edu; 4 Human Nutrition department, Qatar University, Doha 999043, Qatar; zumin.shi@qu.edu.qa

**Keywords:** food safety education, gain- and loss-framed message, personal involvement, medical university students

## Abstract

Objective: This study explored whether the efficacy of food safety education interventions can be increased by message framing among medical university students, and demonstrated the role of personal involvement within the message recipient in moderating framed effects. Methods: A cross-sectional study of food safety message framing was conducted among medical university students (randomly selected 1353 participants). An online self-administered questionnaire was used to collect information. Wilcoxon rank-sum test and Ordered multivariate logistic regression were utilised in the data analyses. Results: The present study showed significant differences in acceptance between the gain- and loss-framed groups (*p* < 0.001). Participants with higher personal involvement had higher acceptance than those with low personal involvement in gain- and loss-framed message models (*p* < 0.001). The acceptance of participants who were concerned about their health condition was higher than those who were neutral regarding their health condition (*p* < 0.001) and participants who suffered a food safety incident had higher acceptance than those who did not (*p* < 0.05). Conclusions: This study portrayed the selection preference of message framing on food safety education among medical university students in southwest China. Participants exposed to loss-framed messages had higher message acceptance than those exposed to gain-framed messages. Personal involvement may affect the food safety message framing. Public health advocates and professionals can use framed messages as a strategy to enhance intervention efficacy in the process of food safety education.

## 1. Introduction

Food safety is a global health goal, and foodborne diseases represent a growing public health problem in developed and developing countries [1], especially those with large populations, such as China [2]. During the period of 2003 to 2008 in China, a total of 2795 foodborne disease outbreaks occurred, which resulted in 62,559 cases, 31,261 hospital admissions, and 330 deaths in 12 provinces of China according to the China National Foodborne Diseases Surveillance Network [3]. Owing to the fact that cases of foodborne diseases were often under-reported, especially in developing countries [4,5], it has been estimated that approximately 200 million cases of foodborne diseases occur in China annually [6]. Furthermore, food safety incidents, such as “milk contaminated with melamine”, “pork products contaminated with clenbuterol”, and “swill-cooked dirty oil” have been frequently reported in recent years [7].

Health education plays a vital role in maintaining and promoting people’s health in China. The State Council of China issued the “Outline of the Plan for Health China 2030” in 2016, which proposed the necessity to establish a sound system of health promotion and education, aiming to improve the capabilities of health education services and popularising scientific knowledge of health [8]. Food safety education is one of the crucial parts of health education, which is of considerable importance to enhance food safety awareness and reduce the risk of foodborne diseases.

However, there are certain limitations in food safety interventions education among university students [9]. Although previous studies demonstrated that conducting food safety education is an important measure to improve the food safety knowledge of university students [10,11], many studies found that university students have inadequate knowledge and inappropriate behaviours regarding food safety, thus placing their health at risk from foodborne diseases [7,12,13,14,15]. Motivating students to receive food safety knowledge and develop favourable awareness and behaviour changes are necessary.

The recent report “Healthy People 2020” published by the US Office of the Surgeon General, detailed the importance of research and evaluation as an aid in the development of these health communication programmes [16]. In accordance with these objectives, researchers have sought to take advantage of the important interplay between theory and practice in designing effective health communication strategies. One consideration for the communication of health information is the framing of the behaviour recommendations and health outcomes in a message [17].

Message framing is theoretically grounded in prospect theory [18], which suggests that people respond differently to information highlighting gains versus losses. If the choices emphasise potential losses, the individual may be generally more willing to choose a risky option to prevent those losses. However, if the choices emphasise potential gains, the individual may be generally less willing to choose options involving risks to secure gains. Drawing on prospect theory, Rothman and Salovey [19] proposed that health messages can be framed in terms of either the benefits of engaging in the recommended behaviour (gain-framed messages) or the costs of not engaging in the behaviour (loss-framed messages). Although conveying essentially identical information, one form of message framing may be more effective at promoting health behaviour change than the other. Many studies also confirmed that framed messages can alter the persuasive impact of a message and profoundly affect choices and behaviours of people [19,20,21]. Specifically, loss-framed messages might be persuasive for illness detection behaviours, such as mammography [22] or skin cancer detection [23], while the gain-framed messages should be more persuasive for illness prevention behaviours, such as physical activity promotion [24]. Differences in persuasiveness exist between gain- and loss-framed messages in some specific behavioural domains. However, earlier meta-analysis found that no statistically significant difference existed in the persuasiveness of gain- and loss-framed appeals for certain domains [25,26], which demonstrated the importance of exploring the role of potential moderators of framing effects.

Investigating the role of potential moderators of framing effects is important to enhance our knowledge of the message framing, which can influence perceptions, attitudes, and behaviours of individuals. Personal involvement has played an important role in the process of responding to health-promoting information [27,28,29]. Previous studies have proposed that framed effects might be used when people are concerned or highly educated regarding a health issue [19,30,31]; these factors may contribute to the systematic processing of information. A limited number of studies explored whether personal involvement moderated message framing effects on food safety, one study indicated that a media food safety story which intended to elicit respondent involvement and a combination of the loss-framed message, was more impactful on the consumer response and willingness to pay for beef products [32]. Furthermore, a previous study has found that concerning food safety issues, thinking about food safety, and having experienced food poisoning were related to food safety behaviours [33]; consumers’ perceived risks of online shopping or dining out would also directly influence the consumption intentions of users [34,35]. Hence, the “To what degree are you concerned about your health condition”, “Food safety incidents experience”, “Dining-out experience”, and “Online food shopping experience” severed as personal involvement in this study.

Medical students’ food safety knowledge, attitudes, and practice are not only important to their health, but are expected to play an important role in health education and promotion after their graduation, which may influence a broader population [36]. The current study was the first that focused on message framing effects on food safety education of medical university students and served personal involvement as potential moderators of food safety message framing. Therefore, this research explored whether the efficacy of food safety education interventions can be increased by message framing among medical university students and demonstrated the role of individual involvement within the message acceptance of moderating framing effects.

## 2. Methods

### 2.1. Study Design and Participants

A cross-sectional study on the acceptance of different modes of food safety message framing was conducted among students in a medical university in Chongqing municipality. The study applied a stratified cluster random sampling method to select the participants. The study randomly sampled four classes out of 38 classes in each grade level, from grade one to three, using a random number table. All the students in the 12 sampled classes were surveyed.

The information was collected through an online survey whilst students were sitting in a computer laboratory at school. During the survey, the researchers were present and answered questions raised by the students. A total of 1353 students participated in the current study. According to the results of the preliminary experiments, the invalid questionnaires, which were defined as a responding time of less than 200 s, were excluded. A total of 1264 students were included in the analysis, and the valid participation rate was 93.4%.

### 2.2. Questionnaire

The questionnaire was self-administered and customised for the target population based on two pilot studies. The first pilot study tested the extent of the message framework description and was completed after several discussions by a panel of experts. Then, the second pilot study investigated 36 students who participated in the pretest to test the reliability of the questionnaire. The Cronbach’s alpha of the questionnaire (demographic characteristics, personal involvement, and framed message materials) was found to be 0.903. The Cronbach’s alpha was found to be 0.861 and 0.903 for the gain-framed and loss-framed message materials, respectively.

The questionnaire had three sections, namely, demographic characteristics, personal involvement variables, and food safety message framing materials. The demographic characteristics included gender, grade, current residence, ethnicity, lack of siblings, and monthly living expenses. The personal involvement variables included four questions: (1) “To what degree are you concerned about your health condition?”; (2) “Did you encounter food safety incidents?”; (3) “How often did you eat out?”; and (4) “How often did you shop for food online?”.

The message framing materials were grouped into three sections based on the food safety knowledge-attitude-practice (KAP) model. Food safety knowledge included three items: (1) “knowledge of storing methods on cooked and raw foods”: gain-framed: “If you know that processing and storing of raw foods and cooked foods should be separated, the risk of cross-contamination, food poisoning, or food-borne illnesses may be reduced”; loss-framed: “If you do not know that processing and storing of raw foods and cooked foods should be separated, the risk of cross-contamination, food poisoning, or foodborne illnesses may be increased”; (2) “knowledge of heating methods on leftovers”; gain-framed: “If you know that cooked food or leftovers should be heated thoroughly, the risk of pathogenic microorganisms or food-borne diseases may be reduced.”; loss-framed: “If you do not know that cooked food or leftovers should be heated thoroughly, the risk of pathogenic microorganisms or food-borne diseases may be increased”; (3) “knowledge of cleaning methods on pesticide residues in fruits and vegetables”; gain-framed: “If you know that fresh vegetable and fruit are treated with water and salt before eating, the risk of pesticide residues may be reduced”; loss-framed: “If you do not know that fresh vegetables and fruit are treated with water and salt before eating, the risk of pesticide residues may be increased”.

Food safety attitude included two items: (1) “attention to food safety events”; gain-framed: “If you have strong intention to pay attention to food safety events in recent years in China, food safety awareness may be improved and the risk of food poisoning or foodborne illness may be reduced”; loss-framed: “If you do not pay attention to food safety events in recent years in China, the risk of food poisoning or foodborne illness may be increased owing to poor food safety awareness”; (2) “intention to change bad eating habits”; gain-framed: “If you develop healthy eating habits (balanced diet; limit salt, cooking oil, added sugar, alcohol, etc.), the risk of related diseases and health problems (overweight, obesity, or other related chronic diseases) may be reduced”; loss-framed: “If you form bad eating habits (unbalanced dietary; excessive salt, cooking oil, added sugar, alcohol, etc.), the risk of related diseases and health problems (overweight, obesity, or other related chronic diseases) may be increased”.

Food safety practice involved seven items: (1) “frying eggs”; gain-framed: “If you fry eggs until the egg-white and egg-yolk are solid, the risk of salmonella infection may be reduced”; loss-framed: “When frying eggs, if you do not wait until egg-white and egg-yolk form a solid, the risk of salmonella infection may be increased”; (2) “dining out”: gain-framed: “If you reduce the frequency of eating out, the risk of overweight and obese may be reduced”; loss-framed: “If you often eat out, the risk of overweight and obese may be increased”; (3) “checking sensory traits”; gain-framed: “When purchasing food, if you pay attention to checking sensory traits of the food (color, smell, aroma, and shape), you may buy fresh and safe food”; loss-framed: “When purchasing food, if you pay no attention to checking sensory traits of the food (color, smell, aroma, and shape), you may buy stale and unsafe food”; (4) “checking food nutrition labels”; gain-framed: “When purchasing foods, if you check nutrition labels (mainly including the nutritional composition table, nutrition claims, and nutritional function claims), dietary balance may be promoted”; loss-framed: “When purchasing food, if you do not check nutrition labels (mainly including nutritional composition table, nutrition claim, and nutritional function claim), the risk of unbalanced diet may be increased”; (5) “checking food labels”; gain-framed: “When purchasing food, if you check the food label (food name, ingredient list, production date, shelf-life, storage conditions, allergenic substances, etc.), high-quality food information may be obtained and the risk of food purchase may be reduced”; loss-framed: “When purchasing food, if you do not check the food label (food name, ingredient list, production date, shelf-life, storage conditions, allergenic substances, etc.), the risk of food purchase may be increased”; (6) “checking online food safety information”; gain-framed: “When shopping for food online, if you check the food safety information, (health permission license, manufacturer, shelf-life, etc.), you may buy safe and high-quality food.”; loss-framed: “When shopping food online, if you do not check the food safety information (health permission license, manufacturer, shelf-life, etc.), you may buy inferior quality food”; (7) “washing hands before handling food”; gain-framed: “If you wash your hands before getting food, the incidence of food contamination or foodborne illnesses may be reduced”; loss-framed: “If you do not wash your hands before getting food, the risk of food contamination or foodborne illness may be increased”. A corresponding photograph was attached to each message to enhance the persuasive effect of messages.

Personal involvement was assessed by four questions: “To what degree are you concerned about your health condition? (1 = not concerned, 2 = neutral, and 3 = concerned)”; “Did you encounter food safety incidents? (1 = yes and 2 = no)”; “How often did you eat out? (1 = never, 2 = occasionally, and 3 = often)”; and “How often did you shop for food online? (1 = never, 2 = occasionally, and 3 = often)”. The scores of personal involvement among participants ranged from 4 to 11, and the number of participants who a score between 4 and 8 was 660 (52.2%). The cut-off was used in order to have a similar proportion in each category; hence, the study defined participants whose scores were below 9 as the low personal involvement group, and scores above 9 as the high personal involvement group.

Food safety message framing materials contained twenty-four questions, each question consisted of five levels with a score ranging from 1 to 5, which implied “totally disagree”, “disagree”, “neutral”, “agree”, and “totally agree”, respectively. The study summarized the scores of the all the items. In the gain-framed model of this study, the score of participants ranged from 32 to 60, and the number of participants who scored below 49, between 49 and 55, and above 55 were 517 (40.9%), 432 (34.2%), and 315 (24.9%), respectively. In the loss-framed model of this study, the score of participants ranged from 33 to 60, and the number of participants who got a score below 49, between 49 and 55, and above 55 were 432 (33.5%), 402 (31.8%), and 439 (34.7%), respectively. Considering the frequency distribution of the score in the gain- and loss-framed models, we defined the below 49 score as the low-score group, a score between 49 and 55 denoted the median-score group and a score above 55 was the high-score group.

### 2.3. Quality Control

The questionnaire was adapted from previous literature [37,38,39,40]. The questionnaire was repeatedly revised through expert interviews and two pilot surveys. The research team members, including teachers and students (postgraduates and undergraduates), all underwent standardised investigation training. Investigators were required to thoroughly understand the approach and methodology of this research, as well as being full of experience in handling potentially sensitive issues. Participants filled out the questionnaires online in the laboratory.

### 2.4. Ethical Approval

All participants provided their informed consent for inclusion before participating in this study. The study protocol was approved by the Ethics Committee of Chongqing Medical University. The record number is 2018011.

### 2.5. Statistical Analysis

The data were carefully processed by Excel software prior to entry into the database. Data analyses were performed using SPSS 20.0 software (IBM Corporation, Armonk, NY, US). The demographic characteristics of participants were expressed as frequencies and percentages. The framework message materials were tested with normality test and homogeneity of variance assumptions. A Wilcoxon signed rank-sum test was performed to assess whether a significant difference existed between gain- and loss-framed messages. The message framing acceptance was expressed as average rank. The factors associated with the framing effects were analysed by Wilcoxon rank-sum test, Wilcoxon signed rank-sum test, and Kruskal–Wallis test. Ordered multivariate logistic regression analysis was implemented to analyse the factors associated with framing effects of food safety. The moderating effects of personal involvement factors were expressed as average rank and high average rank represented by the high acceptance of food safety message framing. All statistics were analysed using a two-sided test. A *p*-value of no more than 0.05 was considered statistically significant.

## 3. Results

### 3.1. Demographic Characteristics of Participants

Demographic characteristics of the participants have been provided in Table 1. The participants comprised of 499 males and 765 females. There were 224 (17.7%) freshman students, 753 (59.65%) sophomore students, and 287 (22.7%) junior students. The Han nationality constituted 89.5% of the sample. There were 634 (50.2%) students from urban areas and 630 (49.8%) from rural areas. The participants with monthly living expenses between 1000 CNY–1499 CNY were 698 (55.2%). Approximately 43.4% of the participants had no siblings.

### 3.2. Analyses of Personal Involvement Variables

As shown in Table 2, more than half (55.8%) of the participants were concerned regarding their health condition and 2.1% were not concerned regarding their health condition among all participants; 4.2% of male and 0.8% of female participants were not concerned regarding their health condition, respectively. In total, 37.2% of all participants encountered food safety incidents. Only 0.6% of all students did not have a dining-out experience. Approximately 9.8% of all participants never shopped for food online, and 15.6% of male and 6% of female participants never shopped for food online.

### 3.3. Analyses of Framed Messages

#### 3.3.1. Test Framing Effects

As shown in Table 3, many food safety messages that used gain-framed and loss-framed descriptions had significant differences (*p* < 0.05) among all participants and female students: (1) Knowledge of heating methods on leftovers; (2) Knowledge of cleaning methods on pesticide residues in fruits; (3) Intention of bad eating habits changes; (4) Frying eggs; (5) Dining out; (6) Checking food sensory traits; (7) Checking food nutrition labels; and (8) Checking food labels. However, several items demonstrated inconsistencies. The messages, which included (1) Knowledge of storing methods on cooked food and raw food; (2) Attention to food safety events; (3) checking food safety information (shelf-life, manufacturer, etc.) during online shopping; and (4) Washing hands before handling food, which showed no significant differences (*p* > 0.05). Regarding male participants, significant differences were observed in the following food safety messages (*p* < 0.05). (1) Knowledge of storing methods on cooked food and raw food; (2) Knowledge of heating methods on leftovers; (3) Intention of bad eating habits changes; (4) Frying eggs; (5) Dining out; (6) Checking food sensory traits; and (7) Checking food labels. Of the food safety messages, which included (1) Knowledge of cleaning methods on pesticide residues in fruits and vegetables; (2) Attention to food safety events; (3) Checking food nutrition labels; (4) Checking food safety information; and (5) Washing hands before handling food, there were no significant differences (*p* > 0.05).

As shown in Table 4, a significant difference was observed between gain- and loss-framed messages (*p* < 0.001). Regarding the gain-framed and loss-framed message model, a significant difference was observed between high personal involvement and low personal involvement (*p* < 0.001). Therefore, those results that demonstrated higher acceptance on the loss-framed than gain-framed food safety messages among participants and among those participants with high personal involvement, had higher acceptance than those who with low personal involvement.

#### 3.3.2. Analyses of Demographic Characteristics and Personal Involvement

Separate moderation analyses were performed to investigate whether the effects of framing on acceptance were moderated by demographic characteristics and personal involvement. Several factors, which included “Gender”, “Grade”, “Residence”, “Ethnicity”, “Lack of sibling”, “Monthly living expenses”, “The degree of concern about health condition”, “Food safety incidents experience”, “Dining-out experience”, and “Online food shopping experience”, were considered.

As shown in Table 5, the results of these analyses indicated that “Gender” (*p* = 0.004 vs. *p* = 0.021), grade (*p* = 0.046 vs. *p* = 0.013), “The degree of concern about your health condition” (*p* = 0.000 vs. *p* = 0.000), and “food safety incidents experience” (*p* = 0.005 vs. *p* = 0.005) exhibited significant differences in gain- and loss-framed messages. The other variables had no significant difference. Compared with males, females had higher rates regarding acceptance of messages (*p* < 0.05). Sophomore had the highest acceptance of messages among all grades (*p* < 0.05). Those participants who were concerned regarding their health condition had the highest rates regarding acceptance of messages than those who felt neutral or provided no attention to their health condition (*p* < 0.05). The participants who suffered a food safety incident had higher acceptance of messages than those who did not suffer any food safety incident (*p* < 0.05).

#### 3.3.3. Ordered Multivariate Logistic Regression Analyses for the Factors Influencing the Message Acceptance

To further investigate the factors that affect the acceptance of food safety messages, the study performed ordered multivariate logistic regression analysis. According to previous studies, “The degree of concern about health condition”, “Food safety incidents experience”, “Gender”, and “Grade” were associated with food safety, which were significant different on the Wilcoxon rank sum test/Kruskal–Wallis test. We chose these four parameters as independent variables.

As shown in Table 6, in the gain-framed message model, compared with participants who were concerned about their health condition, participants who were neutral about their health condition were less likely to exhibit a high acceptance of the message (95% CI (−0.619, −0.193), *p* < 0.001). Participants who did not have food safety incident experience were less likely to gain a high acceptance of message than those who had encountered food safety incidents experience (95% CI (0.034, −0.477), *p* < 0.05).

In the loss-framed message model, compared with participants who were concerned about their health condition, participants who were neutral about their health condition were less likely to gain a high acceptance of message (95% CI (−0.600, −0.179), *p* < 0.001). The participants who did not have food safety incident experience were less likely to have a high acceptance of the message than those who had encountered food safety incidents experience (95% CI (−0.497, −0.07), *p* < 0.05).

## 4. Discussion

This cross-sectional study found a significant difference between gain- and loss-framed food safety messages. Therefore, message framing was effective in the food safety messages of this study. Participants exposed to loss-framed messages showed higher acceptance than those exposed to gain-framed messages. Moreover, the study showed that personal involvement may influence framed messages, and concluded that participants with a higher level of personal involvement produced higher acceptance than those with a lower level of personal involvement.

Empirical studies have demonstrated that gain-framed messages should be more persuasive for health prevention behaviour, and loss-framed messages should be more persuasive for health detection behaviour [22,23,24]. However, this study showed inconsistent results, loss-framed messages were more persuasive than gain-framed messages on the food safety education of medical university students, which is part of the realm of health prevention behaviour. Regarding the gain-framed and loss-framed message model, participants with high personal involvement had higher acceptance than those who with low personal involvement. Several empirical studies were consistent with this study. Riet [41] conducted a test which used anti-smoking framed messages targeted at current smokers. The results found that a loss-framed communication was more persuasive for participants with high self-efficacy to quit smoking than gain-framed communication or no communication. Similarly, Gerend [42] examined the relative effectiveness of gain- versus loss-framed messages in promoting acceptance of a vaccine against human papilloma virus (HPV), and found that a loss-framed message led to considerable HPV vaccination intentions than a gain-framed message only among participants who had multiple sexual partners and those who infrequently used condoms.

Furthermore, this study showed that the participants who were concerned regarding their health condition had the highest acceptance. In addition, participants who suffered food safety incidents had higher acceptance than those who did not. The finding was consistent with previous studies, which proposed that behavioural responses to framed information should be a function of the framed message and pre-existing perceptions of the health issue [43,44]. Particularly, with a health issue experience should influence one's receptivity to information about gain or loss and whether a behaviour is perceived as risky or uncertain to adopt [18,45,46].

Rothman and Salovey [18] proposed that matching the framed message to the type of health behaviour being promoted can increase the persuasiveness of the message. The success of a framed message, which relied on the extent of the perceived risks of the recommended behaviour. Given that people are relatively open to taking risks when faced with potential losses, loss-framed messages should be more effective than gain-framed messages in promoting health detection behaviours. The present study found that food safety acceptance was high when participants were exposed to loss-framed messages (at least among certain individuals). One possible explanation for this finding is that food safety is generally perceived to be a relatively risky behaviour as opposed to a safe one. Similarly, Gallagher [41] found that a loss-framed message may be more effective in people who were perceived with high degree of risk associated with performing health behaviours.

Nevertheless, a few messages, including (1) Knowledge of storing methods on cooked food and raw food; (2) Attention to food safety events; (3) Checking food safety information; and (4) Washing hands before handling food elicited high acceptance using a gain-framed description. The cause of these phenomena may be that such behaviours are often overlooked or considered low perceived risk [47,48,49]. This idea was similar to a recent study [50], which found that gain-framed messages were more effective than loss-framed messages when people perceived low risk. In addition, a previous study [18] proposed that women who were concerned regarding the risk of finding a lump while conducting mammography should be particularly sensitive to a loss-framed appeal. However, when women considered mammography as a health-affirming behaviour, a gain-framed message might more persuasive. Rothman [21] proposed that loss-framed messages were persuasive when people considered a behaviour that they perceived to involve some risks of an unpleasant outcome; by contrast, gain-framed messages were persuasive when people considered a behaviour that they perceived to involve a relatively low risk of an unpleasant outcome.

The present study may confirm that personal involvement plays a crucial role in the food safety message framing effects. “To what degree are you concerned about your health condition” and “food safety incidents experience” might moderate the effects of gain- and loss-framed messages. The degree of personal involvement would influence one’s message processing either in a detailed and integrative manner or in a superficial manner. People who are not considerably involved in or not concerned regarding a certain behaviour are predicted to heuristically process the message [51]. However, high issue involvement led to systematic processing of the message, which favoured loss-framed message [52].

The study was the first to focus on the application of food safety education on message framing, which can broaden the research field of message framework and innovate the education method of food safety. Moreover, to enhance the recognition of message framing among the participants, the current study provided corresponding pictures for each framed message, which supplemented the limitation of previous literature in describing the framed message by plain text.

This study also had certain limitations. Firstly, the use of cross-sectional survey data reduced the researchers’ ability to make direct causal inferences. Secondly, the participants who were investigated only included the medical university students. Hence, the results may not be representative of all university students in China. Further national representative studies are warranted. Thirdly, this study only included personal involvement as a moderator. Future research could consider self-efficacy [20] or emotional state of the message recipient [40] as moderator, which may influence the framed messages. Fourth, we mainly considered the effect of grade on acceptance of the food safety message, but ignored the age of students. Further studies will complete the analysis of demographic characteristics of participants. Finally, the study relied on self-report, which can introduce bias due to dishonesty, over-reporting, under-reporting, and measurement flaws. Limitations of this study would provide interesting avenues for further research.

## 5. Conclusions

This study found that the loss-framed messages received higher acceptance than gain-framed messages on food safety among medical university students. The study also provided support for the contention that personal involvement may influence message framing, which concluded that participants with a high level of personal involvement exhibited higher acceptance of food safety messages. Meanwhile, this study portrayed the selection preference of message framing regarding food safety education among medical university students in southwest China and provided useful information for public health advocates or professionals in the domain of food safety to conduct effective education. Finally, given the enormous growth in the number of food safety incidents over recent years, understanding the promotion of food safety with different framed messages constitutes an important area of food safety education.

## Figures and Tables

**Table 1:** Demographic characteristics of the study population by gender (*n* = 1264).

Variables	Total (*n* = 1264)	Male ( *n*= 499)	Female (*n* = 765)
	*n* (%)	*n* (%)	*n* (%)
Grade			
Freshman	224 (17.7%)	99 (19.8%)	125 (16.3%)
Sophomore	753 (59.6%)	288 (57.7%)	465 (60.8%)
Junior	287 (22.7%)	112 (22.4%)	175 (22.9%)
Residence			
Urban	634 (50.2%)	263 (52.7%)	371 (48.5%)
Rural	630 (49.8%)	236 (47.3%)	394 (51.5%)
Ethnicity			
Han nationality	1131 (89.5%)	443 (88.8%)	688 (89.9%)
Minority	133 (10.5%)	56 (11.2%)	77 (10.1%)
Lack of siblings			
Yes	549 (43.4%)	240 (48.1%)	309 (40.4%)
No	715 (56.6%)	259 (51.9%)	456 (59.6%)
Monthly living expenses			
<¥1000	269 (21.3%)	98 (19.6%)	171 (22.4%)
¥1000–¥1499	698 (55.2%)	280 (56.1%)	418 (54.6%)
¥1500–¥1999	222 (17.6%)	93 (18.6%)	129 (16.9%)
¥2000–¥2499	56 (4.4%)	19 (3.8%)	37 (4.8%)
>¥2500	19 (1.5%)	9 (1.8%)	10 (1.3%)

**Table 2:** Personal involvement of the study population by gender (*n* = 1264).

Variables	Total (*n* = 1264)	Male (*n* = 499)	Female (*n* = 765)
	*n* (%)	*n* (%)	*n* (%)
To what degree are you concerned about your health condition			
Not concerned	27 (2.1%)	21 (4.2%)	6 (0.8%)
Neutral	532 (42.1%)	216 (43.3%)	316 (41.3%)
Concerned	705 (55.8%)	262 (52.5%)	443 (57.9%)
Did you encounter food safety incident			
No	794 (62.8%)	320 (64.1%)	474 (62%)
Yes	470 (37.2%)	179 (35.9%)	291 (38%)
How often did you eat out			
Never	8 (0.6%)	7 (1.4%)	1 (0.1%)
Occasionally	728 (57.6%)	275 (55.1%)	453 (49.2%)
Often	528 (41.8%)	217 (43.5%)	311 (41.7%)
How often did you shop food online			
Never	124 (9.8%)	78 (15.6%)	46 (6%)
Occasionally	859 (68%)	338 (67.3%)	523 (68.4%)
Often	281 (22.2%)	85 (17.0%)	196 (25.6%)

**Table 3:** Analyses of the gain- and loss-framed of food safety messages.

Variables	Total (*n* = 1264)			Male (*n* = 499)			Female (*n* = 765)		
	Gain	Loss	*p*	Gain	Loss	*p*	Gain	Loss	*p*
	Average Rank			Average Rank			Average Rank		
Knowledge									
Knowledge of storing methods on cooked food and raw food	190.8	197.0	0.052	80.23	80.69	0.047 *	117.26	110.35	0.40
Knowledge of heating methods on leftovers	162.9	200.1	0.000 **	66.06	85.09	0.000 **	98.69	115.88	0.000 **
Knowledge of cleaning methods on pesticide residues in fruits and vegetables	134.8	145.2	0.014 *	57.71	65.08	0.623	77.80	79.68	0.003 *
Attitude									
Attention to food safety events	204.5	211.6	0.848	91.13	87.50	0.051	115.75	124.50	0.11
Intention of bad eating habits changes	185.0	179.3	0.000 **	75.39	70.64	0.006 *	110.50	108.86	0.000 **
Practice									
Frying eggs	219.6	218.8	0.000 **	81.20	84.38	0.000 **	138.43	135.44	0.000 **
Dining out	313.4	377.8	0.000 **	133.55	163.75	0.000 **	179.45	214.68	0.000 **
Checking food sensory traits	288.9	343.1	0.000 **	115.82	135.99	0.000 **	172.75	207.73	0.000 **
Checking food nutrition labels	229.6	234.0	0.02 *	88.14	89.89	0.93	142.12	144.30	0.003 *
Checking food labels	232.1	229.1	0.000 **	104.88	104.32	0.000 **	127.84	125.32	0.000 **
Checking food safety information	204.3	201.7	0.986	86.78	84.16	0.631	118.06	117.94	0.671
Washing hands before handling food	159.2	163.7	0.625	67.80	63.34	0.971	91.97	101.03	0.534

Note: Wilcoxon signed rank sum test was used. * *p* < 0.05 and ** *p* < 0.001 (statistically significant).

**Table 4:** Wilcoxon signed rank sum test for testing framed effects.

Variables	Average Rank	Z	*p*
Gain-framed	431.7	−17.748	0.000 **
Loss-framed	559.3		
Gain-framed—low personal involvement	592.3	16.847	0.000 **
Gain-framed—high personal involvement	678.8		
Loss-framed—low personal involvement	591.9		
Loss-framed—high personal involvement	676.8	17.179	0.000 **

Note: Wilcoxon signed rank sum test was used, ** *p* < 0.001(statistically significant).

**Table 5:** Wilcoxon rank sum test/Kruskal–Wallis test for demographic characteristics and personal involvement.

Variables	Gain-Framed				Loss-Framed			
	*n*	Average Rank	*Z/H*	*p*	*n*	Average Rank	*Z/H*	*p*
Gender			−2.876	0.004 *			−2.308	0.021 *
Male	499	596			499	603.26		
Female	765	656.31			765	651.57		
Grade			6.171	0.046 *			8.731	0.013 *
Freshman	224	578.27			224	567.69		
Sophomore	753	646.81			753	648.32		
Junior	287	637.28			287	641.57		
Residence			−1.56	0.119			−1.748	0.08
Urban	634	616.56			634	614.67		
Rural	630	648.54			630	650.45		
Ethnicity			−0.385	0.7			−0.466	0.641
Han nationality	1131	633.85			1131	634.13		
Minority	133	620.99			133	618.61		
Lack of sibling			−1.098	0.272			−1.013	0.311
Yes	549	645.34			549	644.33		
No	715	622.64			715	623.42		
Monthly living expense			0.410	0.982			2.055	0.726
<¥1000	269	630.05			269	623.50		
¥1000–¥1499	698	631.05			698	630.83		
¥1500–¥1999	222	633.30			222	635.63		
¥2000–¥2499	56	622.27			56	695.71		
>¥2500	19	623.21			19	598.32		
To what degree are you concerned about your health condition			25.318	0.000 **			22.531	0.000 **
Not concerned	27	550.20			27	557.06		
Neutral	532	575.97			532	579.14		
Concerned	705	678.31			705	675.65		
Did you encounter food safety incident			−8.152	0.004 *			8.010	0.005 *
No	794	609.98			794	610.22		
Yes	470	670.54			470	670.14		
How often did you eat out			0.065	0.968			0.029	0.986
Never	8	559.81			8	615.50		
Occasionally	728	632.51			728	631.69		
Often	528	632.99			528	633.88		
How often did you shop food online			3.587	0.166			5.702	0.058
Never	124	621.80			124	619.45		
Occasionally	859	622.71			859	619.44		
Often	281	668.81			124	678.19		

Note: Nonparametric test was used. * *p* < 0.05 and ** *p* < 0.001(statistically significant).

**Table 6:** Ordered Multivariate Logistic Regression for demographic characteristics and personal involvement.

Gain-Framed Message						
Parameter		Estimate	SE	95%CI		*p*
Intercept 1		−0.812	0.144	−1.095	0.529	0.000
Intercept 2		0.688	0.144	0.406	0.970	0.000
Gender	Male	−0.207	0.108	−0.419	0.005	0.056
	Female (ref.)					
Grade	Freshman	−0.266	0.167	−0.594	0.061	0.111
	Sophomore	0.024	0.129	−0.230	0.277	0.854
	Junior (ref.)					
To what degree are you concerned about your health condition	Not concerned	−0.342	0.372	−1.071	0.388	0.359
	Neutral	−0.406	0.108	−0.619	0.193	0.000 **
	Concerned (ref.)					
Did you encounter food safety incidents	No	−0.232	0.109	−0.447	−0.018	0.034 *
	Yes (ref.)					
**Loss-Framed Message**						
Intercept 1		−1.185	0.146	−1.471	−0.899	0.000 **
Intercept 2		0.160	0.142	−0.118	0.439	0.259
Gender	Male	−0.151	0.107	−0.361	0.059	0.159
	Female (ref.)					
Grade	Freshman	−0.311	0.165	−0.634	0.013	0.060
	Sophomore	−0.032	0.129	−0.284	0.220	0.803
	Junior (ref.)					
To what degree are you concerned about your health condition	Not concerned	−0.359	0.366	−1.076	0.358	0.326
	Neutral	−0.389	0.107	−0.600	−0.179	0.000 **
	Concerned (ref.)					
Did you encounter food safety incidents	No	−0.283	0.109	−0.497	−0.070	0.009 *
	Yes (ref.)					

Note: Ordered multivariate logistic regression analysis. * *p* < 0.05 and ** *p* < 0.001 (statistically significant).
